# Changes in Chinese college students’ mobile phone addiction over recent decade: The perspective of cross-temporal meta-analysis

**DOI:** 10.1016/j.heliyon.2024.e32327

**Published:** 2024-06-03

**Authors:** Chunwei Lyu, Zixuan Cao, Zun Jiao

**Affiliations:** a School of Educational Studies, Universiti Sains Malaysia, Penang, Malaysia; b Institute of Heath Management and Policy, School of Public Policy and Administration, Xi'an Jiaotong University, Xi'an, China; c City Graduate School, City University of Malaysia, Petaling Jaya, Malaysia

**Keywords:** Mobile phone addiction, Chinese college students, Changes, Recent decade, Cross-temporal meta-analysis

## Abstract

In recent years, with the rapid advancement of Internet technology and the impact of the COVID-19 pandemic, mobile phones have been used more frequently, the development trend of mobile phone addiction among Chinese college students is a concern to society. This cross-temporal meta-analysis provides compelling evidence of the rising trend of mobile phone addiction in Chinese college students based on data from 42 independent studies (Sample Size = 49,544) over the past decade (2013–2022), and discuss the three important rising periods. Furthermore, extended research has revealed correlated factors of mobile phone addiction among Chinese college students, including gender, anxiety, depression, loneliness, stress, well-being, social support, and resilience. Additionally, the study identified the National internet penetration rate and the National GDP index as significant predictors of mobile phone addiction in Chinese college students. These findings not only reveal the changing trends of mobile phone addiction among Chinese college students, but also enrich the understanding of mobile phone addiction among this population and provide a reference for improving mobile phone addiction among in the future.

## 1 Introduction

The swift evolution of internet technology has spurred the growth of the mobile phone industry, establishing it as an essential tool in the fabric of modern daily life within contemporary society. According to the 51st Statistical Report on Internet Development in China, as of December 2022, mobile phone Internet users in China reached 1.065 billion, representing 99.8 % of all Internet users [1], has far surpassed other electronic end devices such as laptops, tablets, and desktop computers. While mobile phones undeniably enhance convenience in daily activities, frequent and excessive usage can lead to addiction [2]. Mobile phone addiction is an extreme dependence on and excessive use of mobile phones by individuals to the extent that it negatively affects their daily life, work, and social life [3,4], as well as their physical and mental health [5]. Furthermore, due to the widespread impact of the COVID-19 pandemic, offline activities have been severely limited during this period, leading to more frequent use of mobile phones, addiction to mobile phones has emerged as a societal public health issue that impedes the healthy growth of adolescents [5].

Excessive or frequent mobile phone use can result in physical symptoms, including eye fatigue, hearing loss, arm numbness, swelling of wrist joints, and neck pain [6]. Mobile phone addiction also detrimentally impacts academic performance, and overall quality of life [7], and contributes to health-risk behaviors [8], such as smoking initiation, sleep disorders, and inadequate physical exercise. With the COVID-19 pandemic sweeping China, a large amount of study time has shifted to online for students, which has led to the heavy use of electronic tools, making it easier to induce mobile phone addiction. A study conducted in 2021 suggests that the prevalence of severe mobile phone addiction among Chinese college students is serious at over 25 % [9], In addition, the state of mobile phone addiction among Chinese college students has changed over time [10,11], and there is still a lack of conclusive evidence to identify change trends in mobile phone addiction among Chinese college students.

On the other hand, addressing the problem of mobile phone addiction has become imperative [12], so it is necessary to actively explore the potential causes and mechanisms of mobile phone addiction in Chinese college students. Firstly, numerous previous studies have shown gender differences in mobile phone addiction among college students in China [13,14], but some studies have proven that gender and phone addiction are not significantly related [15,16]. Secondly, to explore the influencing factors of mobile phone addiction, academics have conducted research from individual, family, and social perspectives showing that negative emotions such as anxiety [17], depression [18], and loneliness [19] can lead to mobile phone addiction, but the specific effect sizes are not yet clear. Finally, a correlation seems to exist between mobile phone addiction among college students and social development in China [20], but the exact relationship remains unclear. Consequently, another objective of this study was to explore the factors associated with mobile phone addiction among Chinese college students.

As of yet, no research has been able to present a comprehensive evaluation of the change trends of mobile phone addiction among Chinese college students. The meta-analysis can instead of the potential sampling or measurement deficiencies inherent in a single study [21], provide a solution to this issue by combining numerous empirical surveys through a mathematical model for evaluation. Therefore, according to the study objectives, this study conducted a cross-temporal meta-analysis based on the Mobile Phone Addiction Index (MPAI), comprehensively analyzing the development trend of mobile phone addiction and the correlated factors among Chinese college students over the past decade. The Mobile Phone Addiction Index (MPAI) is the most extensive instrument in the history of mobile phone addiction research among Chinese college students, it has two Chinese versions [22,23], both featuring identical content and structure.

## 2 Method

The Preferred Reporting Items for Systematic Reviews and Meta-Analyses (PRISMA) guidelines [24] and Cochrane guidelines for performing systematic reviews [25] informed the execution of the present meta-analysis. The protocol for this meta-analysis was registered in PROSPERO (PROSPERO: CRD42023482227).

### 2.1 Search strategy

The included studies were restricted to English articles published in peer-reviewed journals from three international databases (Web of Science, Scopus, and PubMed), as well as peer-reviewed Chinese articles from three mainstream Chinese databases (CNKI, WANFANG, and VIP). Remarkably, to ensure the quality of the included studies, only core journal articles from the three Chinese databases were selected, including the PKU Core Journals, the Chinese Science Citation Database (CSCD), and the Chinese Social Sciences Citation Index (CSSCI). In addition, this study manually searched Google Scholar and Baidu Scholar, expecting to incorporate as many of the studies that were not already available in each database as possible. The search years were limited from January 1, 2014, to January 1, 2024, with the search string (“Mobile Phone Addiction Index” OR “MPAI”) AND (“Chinese College Students” OR “Chinese University Students” OR “Chinese Undergraduates”). The Endnote software was used to import all articles searched and screen them.

### 2.2 Inclusion and exclusion criteria

The included criteria in the present meta-analysis: 1) Studies must be published in peer-reviewed journals in English or Chinese; 2) Measurement of mobile phone addiction levels must utilize the Mobile Phone Addiction Index (MPAI); 3) Participants must all be Chinese college students; 4) Full text available, and explicit quantitative indicator data reported (Sample Size, Mean and SD of MPAI); 5) Studies must be published between January 1, 2014, and January 1, 2024.

If any following criteria are met, the study will be excluded: 1) Studies published in languages other than English and Chinese; 2) Studies measuring mobile phone addiction using instruments other than the MPAI; 3) Participants were not Chinese college students; 4) Full text missing or data missing; 5) Studies published before January 1, 2014 or after January 1, 2024; 6) The study didn't make prominent scholarly contributions (such as Chinese non-core journals) or were considered low quality in Quality Assessment.

### 2.3 Screening and data extraction

Following the import of all the retrieved studies into the Endnote software and eliminating the duplicates, check and screen each study's title, abstract, and full text according to the inclusion and exclusion criteria for the screening. After the screening, the data required for this meta-analysis were extracted from the included studies, including authors, year of publication, data collected year, sample detail, and data outcomes (Mean and SD of MPAI). Regarding the data collected year, based on the previous experience with cross-temporal meta-analysis, if the year of data collection is reported in the text, the study will be coded using the reported year as the data collected year, if the article wasn't reported, the data collected year was coded as two years before publication [26]. Two authors will first independently screen and extract the study data, if disagree, a third author will decide.

### 2.4 Quality assessment

Using the JBI Critical Appraisal Checklist for Analytical Cross Sectional Studies to evaluate the quality of included studies [27]. The JBI tool consists of eight items covering aspects of study design, methodology, results and conclusions to ensure the reliability and validity of the study (1. Were the criteria for inclusion in the sample clearly defined? 2. Were the study subjects and the setting described in detail? 3. Was the exposure measured in a valid and reliable way? 4. Were objective, standard criteria used for measurement of the condition? 5. Were confounding factors identified? 6. Were strategies to deal with confounding factors stated? 7. Were the outcomes measured in a valid and reliable way? 8. Was appropriate statistical analysis used?), items 3 and 4 are not applicable and excluded in this study [28,29]. The assessment criteria are scores equal to or lower than 49 % considered Low quality, scores from 50 % to 69 % considered Medium quality, and scores equal to or above 70 % considered High quality [30], all studies were evaluated independently by two authors and if there were disagreements, a resolution was invited to a third author.

### 2.5 Statistical analysis

The statistical analysis of the data was conducted with SPSS 25.0 and STATA 17. First, a scatterplot of the MPAI scores versus the data collected year was made using the mean MPAI scores from each included studies, and a line graph versus the MPAI weighted mean score and the data collected year, the total MPAI mean score over the years was weighted by the sample size of each study (Formula:‾x = ∑ x_i_ × n_i_/∑ n_i_), unfold the change trend of mobile phone addiction in Chinese college students in the past decade.

Secondly, Pearson's correlation analysis of MPAI scores with the data collected year provided the basis for the regressions. While weighting the sample size, a simple linear regression analysis was conducted with the year as the independent variable and the MPAI score as the dependent variable. A regression equation was used to examine the relationship between year and mobile phone addiction among Chinese college students, and calculated the effect size (Cohen's d) to measure the amount of change in mobile phone addiction among Chinese college students over the past decade (The Cohen's d is calculated as follows: Substituting the first year (2013) and last year (2022) into the resulting regression equation yields the starting and ending year MPAI mean score. The difference between the mean score of the starting year and the mean of the ending year divided by the total mean standard deviation of the years is the value of Cohen's d).

Thirdly, to explore the factors correlated with mobile phone addiction among Chinese college students, a meta-analysis was conducted on mobile phone addiction among male and female college students, and since the measurement tools of the studies were all the Mobile Phone Addiction Index (MPAI), employed the Weighted Mean Difference (WMD) [31]. In addition, this study will count all correlated variables related to mobile phone addiction among Chinese college students in the included studies, correlated variables with an appearance frequency of more than two were selected and combined correlation coefficients to reveal which variables were most closely related to mobile phone addiction among Chinese college students and evaluate the level of correlation, used the Fisher's Z as the effect size because the indicators combined were correlation coefficients (Fisher’ Z = 0.5 × ln [(1 + r)/(1 - r)]). The χ^2^ test was used to ascertain the presence of significant heterogeneity among the studies, A fixed-effects model was selected if P > 0.1 and I^2^ < 50 % indicated that there was acceptable heterogeneity between the studies; a random-effects model was selected if P < 0.1 and I^2^ > 50 % indicated that there was heterogeneity between the studies [32], and the publication bias was evaluated by Egger's test [33].

Finally, the National internet penetration rate and the National GDP index, were selected social indicators, to examine the role of social development in predicting the level of mobile phone addiction among Chinese college students using regression analysis and lagged regression analysis. The social indicators are derived from each annual Statistical Report on Internet Development in China published by the China Internet Network Information Center (CNNIC) and the China Statistical Yearbook for the year 2023 released by the National Bureau of Statistics of China (NBS).

## 3 Results

### 3.1 Search results

**Fig. 1:**
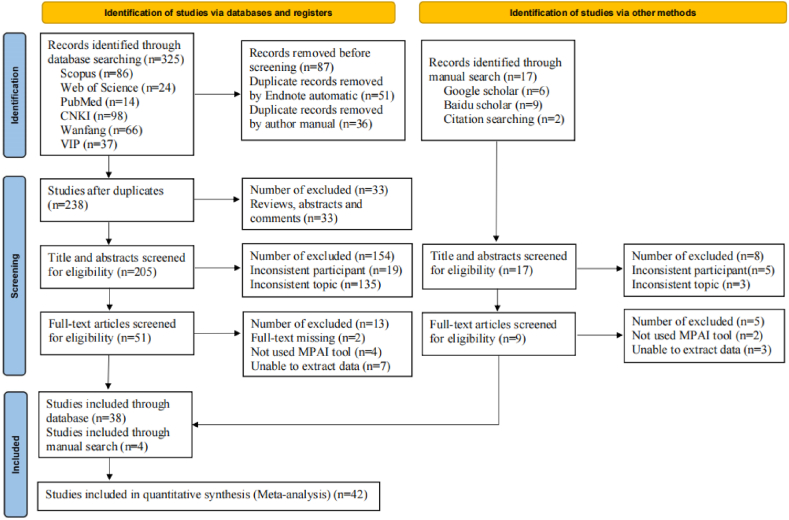
Flow diagram for included studies.

According to the search strategy, six databases yielded 325 studies (Fig. 1). With the Endnote software automatic and author manual searches, 87 duplicate studies were excluded, and 238 were retained. During the screening phase, 33 studies with non-journal article types were eliminated, back then according to the inclusion and exclusion criteria, 154 and 13 each by browsing through the abstracts and full text, thus 38 studies were included through the database search. In addition, two scholarly websites were utilized for the manual search to prevent missing any relevant literature, 13 studies were excluded from the 17 articles that were obtained by applying the same screening as the database, therefore, through the manual search, 4 studies were included. Finally, 42 studies were included in this meta-analysis.

### 3.2 Description of studies

Table 1 summarizes the information of all 42 studies included in the present meta-analysis, 15 in English and 27 in Chinese. All participants are Chinese college students aged 18–24 years old, with a total sample size of 49,544, about 44.87 % male students. All studies used cross-sectional surveys and reported mean scores and standard deviations for the mobile phone addiction index (MPAI).

**Table 1:** Information of the included studies.

Author (Year)	Data Year	N	Age	% Male	Mean	SD
Huang, Niu et al. (2014)^b^ [22]	2013	1392	19.28	57.26 %	36.54	11.48
Huang, Hou et al. (2014)^b^ [34]	2013	1172	19.95	58.02 %	35.03	12.84
Wang et al. (2014)^b^ [17]	2013	493	18–24	43.81 %	40.68	11.22
Liu and Cai (2015)^b^ [35]	2013	328	18.30	35.37 %	37.65	10.93
Wang and Zhang (2015)^b^ [36]	2014	3738	20.00	34.40 %	38.84	11.14
Zhang et al. (2015)^b^ [37]	2014	368	18–23	47.83 %	42.60	12.20
Deng et al. (2015)^b^ [38]	2014	1477	19.00	56.26 %	36.80	11.50
Huang et al. (2015)^b^ [39]	2014	2514	19.23	56.05 %	35.06	11.39
Yao et al. (2016)^b^ [40]	2014	418	19.85	47.13 %	42.76	10.14
Lian et al. (2016)^a^ [41]	2015	742	19.14	55.39 %	44.88	11.39
Zhou et al. (2017)^b^ [42]	2015	619	20.30	46.53 %	41.12	11.69
Hu et al. (2017)^b^ [43]	2016	1950	19.14	57.49 %	37.97	11.62
Ni and Deng (2017)^b^ [44]	2016	612	18–22	43.63 %	39.09	10.84
Zhang et al. (2018)^b^ [45]	2016	732	18–23	40.44 %	45.17	9.86
Gao et al. (2018)^b^ [46]	2017	360	18–23	46.11 %	43.25	11.59
Chen et al. (2018)^b^ [16]	2017	1912	19.66	36.82 %	43.22	11.20
Zhao (2019)^b^ [47]	2017	362	18–24	43.09 %	43.22	12.22
Hao et al. (2019)^a^ [10]	2018	847	20.13	51.24 %	41.85	11.62
Zhu et al. (2019)^a^ [48]	2018	356	18.33	36.30 %	42.67	10.88
Xie and Song (2019)^b^ [49]	2018	493	22.00	12.98 %	43.05	10.10
W. F. Li et al. (2020)^a^ [50]	2018	345	19.75	37.10 %	42.73	11.11
Chen et al. (2020)^b^ [51]	2018	578	19.70	39.79 %	41.39	12.10
Qiu et al. (2020)^b^ [52]	2018	1962	18–23	38.84 %	41.45	10.88
Zhao et al. (2021)^a^ [53]	2018	1123	18–22	37.31 %	48.62	10.88
L. Li et al. (2020)^a^ [54]	2019	1164	20.10	43.47 %	42.09	10.24
Yang et al. (2020)^b^ [55]	2019	874	19.54	23.11 %	47.60	12.41
Li et al. (2021)^a^ [56]	2019	1078	20.00	71.61 %	48.28	11.39
Liu et al. (2021)^a^ [57]	2019	908	21.04	47.80 %	46.24	14.11
Zhao et al. (2022)^a^ [58]	2019	545	19.59	19.82 %	43.34	10.99
Chen et al. (2021)^b^ [59]	2020	1339	19.68	30.47 %	41.71	10.31
Huang et al. (2021)^b^ [60]	2020	838	18–24	58.35 %	53.72	10.07
Li et al. (2022)^a^ [61]	2020	1258	20.20	39.90 %	42.15	10.24
Wang and Wang (2022)^b^ [62]	2020	1356	18.57	53.24 %	44.48	11.97
Jiang et al. (2022)^a^ [63]	2021	1491	20.80	73.31 %	38.12	13.33
Liu et al. (2022)^a^ [64]	2021	1129	19.82	56.24 %	45.73	11.90
Li and Long (2022)^b^ [65]	2021	1454	17–24	52.68 %	49.97	3.38
Wang et al. (2023)^a^ [14]	2021	1112	21.40	38.94 %	45.70	6.8
Cheng et al. (2023)^b^ [66]	2021	521	18.82	41.65 %	45.18	11.32
Jiang et al. (2023)^a^ [67]	2022	354	19.30	50.30 %	44.99	9.94
Xie et al. (2023)^a^ [13]	2022	7234	18.86	29.82 %	45.00	13.59
Wei (2023)^b^ [68]	2022	838	20.70	66.83 %	65.11	7.82
Zhang et al. (2023)^b^ [69]	2022	1158	18.26	27.72 %	46.11	11.92

Note.

a English article.

b Chinese article; Date Year = Data collection year; N = Number of participants; Mean = Mean score; SD = Standard deviation.

### 3.3 Results of quality assessment

The included studies have generally good quality (Table 2). Using the criteria outlined in the JBI tool [27], eight items in total, items 3 and 4 of the JBI tool were not applicable and were excluded here. Sampling is clearly defined. Although the participant's ages are reported to vary, the information about each participant is described in detail, all participants are Chinese college students. Valid and objective measures were employed in all studies. All studies performed appropriate statistical analyses, but fewer took confounding factors into account or employed strategies to address them. All studies reported the mean scores and standard deviations of MPAI.

**Table 2:** Quality assessment using the JBI tool.

Author (Year)	1	2	5	6	7	8	Quality
Huang, Niu et al. (2014) [22]	Yes	Yes	No	No	Yes	Yes	Medium
Huang, Hou et al. (2014) [34]	Yes	Yes	No	No	Yes	Yes	Medium
Wang et al. (2014) [17]	Yes	Yes	No	No	Yes	Yes	Medium
Liu and Cai (2015) [35]	Yes	Yes	No	No	Yes	Yes	Medium
Wang and Zhang (2015) [36]	Yes	Yes	Yes	Yes	Yes	Yes	High
Zhang et al. (2015) [37]	Yes	Yes	Yes	Yes	Yes	Yes	High
Deng et al. (2015) [38]	Yes	Yes	Yes	Yes	Yes	Yes	High
Huang et al. (2015) [39]	Yes	Yes	Yes	Yes	Yes	Yes	High
Yao et al. (2016) [40]	Yes	Yes	Yes	Yes	Yes	Yes	High
Lian et al. (2016) [41]	Yes	Yes	No	No	Yes	Yes	Medium
Zhou et al. (2017) [42]	Yes	Yes	No	No	Yes	Yes	Medium
Hu et al. (2017) [43]	Yes	Yes	No	No	Yes	Yes	Medium
Ni and Deng (2017) [44]	Yes	Yes	No	No	Yes	Yes	Medium
Zhang et al. (2018) [45]	Yes	Yes	No	No	Yes	Yes	Medium
Gao et al. (2018) [46]	Yes	Yes	No	No	Yes	Yes	Medium
Chen et al. (2018) [16]	Yes	Yes	No	No	Yes	Yes	Medium
Zhao (2019) [47]	Yes	Yes	No	No	Yes	Yes	Medium
Hao et al. (2019) [10]	Yes	Yes	No	No	Yes	Yes	Medium
Zhu et al. (2019) [48]	Yes	Yes	Yes	Yes	Yes	Yes	High
Xie and Song (2019) [49]	Yes	Yes	No	No	Yes	Yes	Medium
W. F. Li et al. (2020) [50]	Yes	Yes	No	No	Yes	Yes	Medium
Chen et al. (2020) [51]	Yes	Yes	No	No	Yes	Yes	Medium
Qiu et al. (2020) [52]	Yes	Yes	Yes	Yes	Yes	Yes	High
Zhao et al. (2021) [53]	Yes	Yes	No	No	Yes	Yes	Medium
L. Li et al. (2020) [54]	Yes	Yes	No	No	Yes	Yes	Medium
Yang et al. (2020) [55]	Yes	Yes	Yes	Yes	Yes	Yes	High
Li et al. (2021) [56]	Yes	Yes	Yes	Yes	Yes	Yes	High
Liu et al. (2021) [57]	Yes	Yes	Yes	Yes	Yes	Yes	High
Zhao et al. (2022) [58]	Yes	Yes	No	No	Yes	Yes	Medium
Chen et al. (2021) [59]	Yes	Yes	No	No	Yes	Yes	Medium
Huang et al. (2021) [60]	Yes	Yes	Yes	Yes	Yes	Yes	High
Li et al. (2022) [61]	Yes	Yes	Yes	Yes	Yes	Yes	High
Wang and Wang (2022) [62]	Yes	Yes	No	No	Yes	Yes	Medium
Jiang et al. (2022) [63]	Yes	Yes	No	No	Yes	Yes	Medium
Liu et al. (2022) [64]	Yes	Yes	No	No	Yes	Yes	Medium
Li and Long (2022) [65]	Yes	Yes	No	No	Yes	Yes	Medium
Wang et al. (2023) [14]	Yes	Yes	Yes	Yes	Yes	Yes	High
Cheng et al. (2023) [66]	Yes	Yes	No	No	Yes	Yes	Medium
Jiang et al. (2023) [67]	Yes	Yes	No	No	Yes	Yes	Medium
Xie et al. (2023) [13]	Yes	Yes	No	No	Yes	Yes	Medium
Wei (2023) [68]	Yes	Yes	Yes	Yes	Yes	Yes	High
Zhang et al. (2023) [69]	Yes	Yes	No	No	Yes	Yes	Medium

Note: Scores equal or below 49 % considered Low quality; Scores from 50 % to 69 % considered Medium quality; Scores equal or above 70 % considered High quality.

### 3.4 Results of statistical analysis

#### 3.4.1 Trends in Chinese college students’ mobile phone addiction over year

**Fig. 2:**
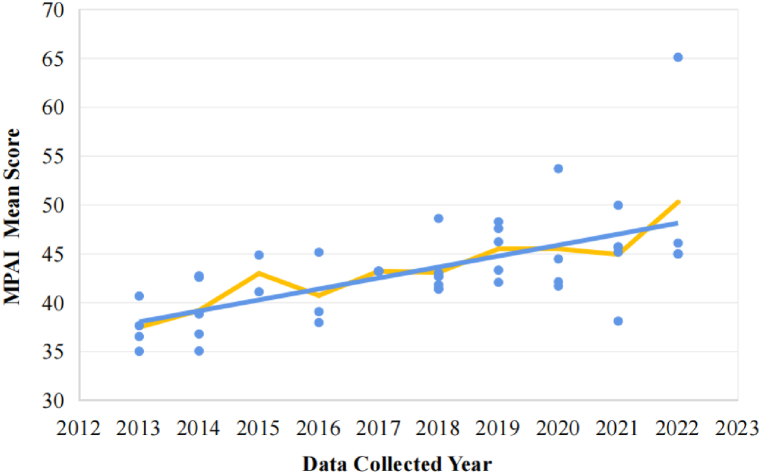
Scatterplot of MPAI mean scores and data collected year.

**Fig. 3:**
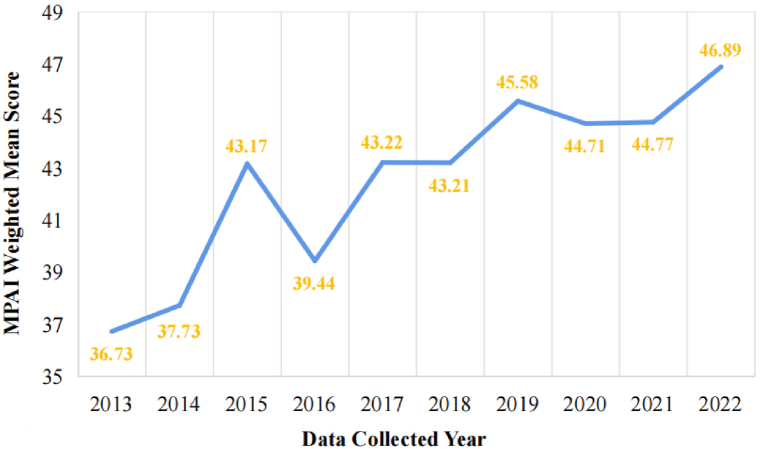
Line graph of MPAI weighted mean scores and data collected year.

The scatterplot (Fig. 2) based on the MPAI mean scores reported by the included studies and the data collected year shows that the level of mobile phone addiction in Chinese college students has been increasing over the years. The line graph further validates this finding (Fig. 3), which is drafted by weighting the sample size and calculating the total MPAI weighted mean score over each year.

A substantial correlation was found from Pearson's correlation analysis results between the mobile phone addiction index (MPAI) and the data collected year (r = 0.618, P = 0.000), allowing for the next step of regression analysis. Regression analysis was conducted while weighting the sample size, with the data collected year as the independent variable and the MPAI score as the dependent variable. The results of regression analysis (Table 3) revealed that the data collected year significantly predicted the MPAI score after controlling for sample size (β = 0.660, P = 0.000). The regression equation: MPAI Score = 1.078 × Data Collected Year - 2133.467.

**Table 3:** Regression analysis results.

Predictor	Unstandardized		Standardized	Unstandardized 95 % CI	R^2^
	b	SE	β		
Intercept	−2133.467	391.253		[-2924.220, −1342.715]	0.422
Year	1.078	0.194	0.660^a^	[0.687, 1.470]	

Note: Regression weighted by sample size.

a P < 0.001; β = Regression coefficient.

Substituting the start year (2013) and the end year (2022) into the regression equation yields the start year MPAI mean score (M_2013_ = 36.55) and the end year MPAI mean score (M_2022_ = 46.25). The results show a 9.7-point increase in the MPAI score from 2013 to 2022, the effect size of Cohen's d (d = 0.88) was a high degree, indicating a large magnitude of change (Table 4).

**Table 4:** Change in mobile phone addiction.

	M_2013_	M_2022_	M_Difference_	M_SD_	Cohen's d
MPAI	36.55	46.25	9.70	11.01	0.88

Note: MPAI = Mobile Phone Addiction Index; M_2013_ = Start year mean score; M_2022_ = End year mean score; M_Difference_ = M_2022_ - M_2013_; M_SD_ = Mean Standard Deviation of MPAI over 10 years; Cohen's d = M_Difference_/M_SD_.

#### 3.4.2 Factors related to Chinese college students’ mobile phone addiction

**Fig. 4:**
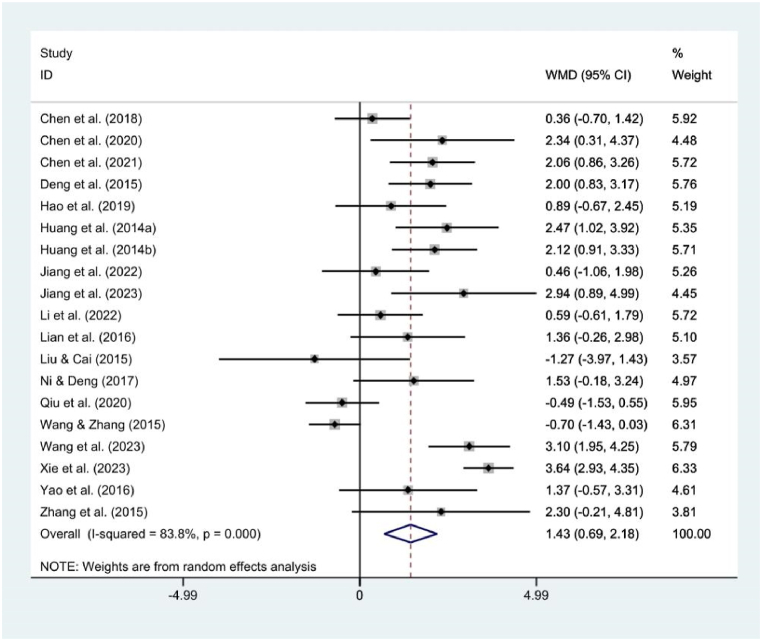
Forest plot of gender difference in MPAI among Chinese college student.

Fig. 4 illustrates the gender difference in mobile phone addiction among college students in China. Due to the higher heterogeneity between studies, a random effects model was used, and the results of the meta-analysis demonstrated that female college students' MPAI score was higher than male college students (WMD = 1.43, 95 % CI [0.69, 2.18], P = 0.000), indicates that mobile phone addiction is more serious among Chinese female college students than males. The *P*-value of Egger's test was 0.968 (P > 0.05), indicating that there was no risk of publication bias.

As shown in Table 5, after screening, seven of the variables correlated with mobile phone addiction among college students in China appeared more than two times in the included studies. The results of the χ^2^ test showed that random effects models were used for all variables except depression (I^2^ = 0.0 %, P = 0.979) and resilience (I^2^ = 0.0 %, P = 0.811) heterogeneity, which were low between studies and used fixed effects models. The results of the meta-analysis showed that anxiety (r = 0.37), depression (r = 0.42), loneliness (r = 0.32), and stress (r = 0.41) were positively correlated with mobile phone addiction among Chinese college students, while well-being (r = −0.30), social support (r = −0.12), and resilience (r = −0.13) were negatively correlated with mobile phone addiction in Chinese college students. All of Egger's test p-values were >0.05, indicating that there was no publication bias.

**Table 5:** Correlation between influence factors and mobile phone addiction.

Influence factor	Z	95 % CI	I^2^ (%)	r	K	N	P
Anxiety	0.39	[0.34,0.44]	79.6 %	0.37	10	7874	0.515
Depression	0.45	[0.42,0.48]	0.0 %	0.42	5	5067	0.257
Loneliness	0.33	[0.27,0.38]	58.8 %	0.32	5	3100	0.222
Stress	0.44	[0.32,0.56]	88.7 %	0.41	3	2575	0.478
Well-being	−0.31	[-0.53,-0.10]	98.7 %	−0.30	3	7127	0.296
Social support	−0.12	[-0.19,-0.06]	67.7 %	−0.12	3	5189	0.564
Resilience	−0.13	[-0.16,-0.11]	0.0 %	−0.13	3	8172	0.627

Notes: Z = Fisher's Z; r = Summary Pearson's correlation coefficient; K=Number of studies; N = Sample size; P = Egger's test result.

#### 3.4.3 Social indicators predict Chinese college students’ mobile phone addiction

Table 6 provides the relationship of both the National internet penetration rate and the National GDP index of five years prior, three year prior, and during the year of data collection with Chinese college students’ mobile phone addiction. The results of regression analysis of MPAI among Chinese university students using the National internet penetration rate and the National GDP index as independent variables were statistically significant, especially at a lag of three and five years, still showed significant, indicating that the enhancement in mobile phone addiction among Chinese college students is related to these two social indicators, the National internet penetration rate and the National GDP index would significantly predict mobile phone addiction among college students in China.

**Table 6:** Relation between the societal indicators and Chinese college students’ MPAI.

Societal Indicators	Five years prior		Two years prior		Current year	
	b	β	b	β	b	β
Internet penetration rate	0.290^b^	0.916	0.323^b^	0.894	0.273^a^	0.860
GDP index	1.128^b^	0.897	1.146^b^	0.883	0.984^a^	0.843

Note: *P < 0.05.

a P < 0.01.

b P < 0.001; b = Standardized regression coefficient; β = Standardized regression coefficient.

## 4 Discussion

In recent years, several scholars have argued that mobile phone addiction among Chinese college students seems to be getting worse [10,11], this study provides evidence for this view based on a cross-temporal meta-analysis perspective. This meta-analysis included 42 studies, with a sample size of 49,544 in total and a period of 10 years (2013–2022). The results show that the mobile phone addiction status in Chinese college students has been rising over time, and the MPAI score increased from 36.55 in 2012 to 46.25 in 2022, with a large effect size of change (d = 0.88).

Furthermore, as shown in Fig. 2, Fig. 3, the rising trend of mobile phone addiction among Chinese college students in the decade can be divided into three important rising periods. The first is the explosive growth around 2015, which may be related to the craze for mobile games among young people, lot of research indicates that highly significant correlation between mobile phone addiction and online gaming addiction [70,71]. Additionally, the release of Honor of Kings, one of the most popular mobile games in recent years [72], has become a significant catalyst. As a mobile online game, minors under the age of 18 are only allowed to log in for 2 h a day by national laws, which has led to the fact that college students are the main players of Honor of Kings [73]. Secondly, there was a noticeable upward trend around 2017, which may be closely correlated with the popularity of short videos, particularly in the adolescent demographic, apps like TikTok became essential [74]. According to statistics, Chinese college students spend the most time on daily has shifted from social networking apps to short video apps [75,76], also with a significant increase in usage time. Finally, around 2019, there was a rising in mobile phone addiction among Chinese college students, which may be related to the isolation policies during the COVID-19 pandemic. To prevent the spread of COVID-19, the Chinese government implemented a series of isolation policies, resulting in the majority of college courses being moved online. This substantial increase in the use of mobile internet devices significantly contributed to mobile phone addiction among Chinese college students [7].

Building upon above foundation, this study delves into the factors correlated with mobile phone addiction in Chinese college students during the past decade, as well as the societal indicators that impact this phenomenon. Firstly, the findings of the meta-analysis based on gender showed that female college students’ mobile phone addiction was more serious than male (WMD = 1.43, 95 % CI [0.69, 2.18], P = 0.000). This discrepancy is may attributed to notable gender differences in internet usage preferences [77], with males gravitating towards online gaming, while females exhibit preferences for online interpersonal communication and shopping [78,79]. Over the past decade, China has witnessed a burgeoning social interaction industry, with female college students dedicating significant time to engaging with friends, family, and online acquaintances through social apps [80], concurrently, as well as the rapid evolution of the online shopping sector, female college students serve as the primary consumer force, substantial time investments are directed toward online shopping activities [81], the basic vehicle for these online behaviors is the mobile phone. Conversely, male college students appear to prefer activating their computers and engaging in gaming activities rather than utilizing mobile phones for interpersonal interactions or browsing for shopping information [81,82], which reinforces the fact that female college students in China are more dependent on their mobile phones than male. Additionally, when choosing college majors, males tend to prefer STEM fields, while females tend to prefer majors in humanities and social sciences [83]. This difference in majors may also lead to differences in the patterns and preferences of technology usage between male and female college student [84], thereby result the gender differences in mobile phone addiction among college students.

Secondly, anxiety (r = 0.37), depression (r = 0.42), loneliness (r = 0.32), and stress (r = 0.41), along with well-being (r = −0.30), social support (r = −0.12), and resilience (r = −0.13), have all been identified as influential factors in the development of mobile phone addiction among college students in China. Previous research has consistently demonstrated that negative emotions, such as anxiety, depression, stress, and loneliness, can significantly contribute to mobile phone addiction [57,85]. This correlation is explained by the escapism mechanism, college students may resort to mobile phone usage as a means to escape or temporarily alleviate negative emotions in reality, seeking short-term psychological comfort or entertainment [86]. In addition, low levels of well-being and social support signify a deficiency in emotional interaction with others [87], while a lack of resilience results in inadequate coping strategies [88]. In response, college students may turn to mobile phones to fill emotional voids and compensate for social deficits. However, it is important to note that mobile phone addiction as an escapist behavioral pattern, offers only transient psychological comfort [89], in the long term may establish an unhealthy coping pattern, contributing to a vicious cycle where negative emotions drive individuals to seek relief through mobile phone use, mobile phone addiction can conversely exacerbate these negative emotions [19,90].

Finally, based on officially published data, this study identifies that the National internet penetration rate and the National GDP index will significantly predict the rise of mobile phone addiction in Chinese college students. According to the investigation, the Internet has the highest penetration rate among students in China [91]. The widespread availability of the internet has enriched the functionalities of mobile phones, enabling college students to address various daily needs through their mobile phones. As mobile phone usage becomes ingrained as a habit, the inevitable consequence is the development of psychological dependence, leading to mobile phone addiction [2]. In addition, previous studies have found that is mobile phone addiction symptoms associated with socioeconomic level [92], and the results of the present study support this view. According to the theory of social change and human development, as a nation's economic level ascends, college students may grapple with challenges in swiftly adapting to the ensuing social transformation. This struggle in adaptation can result in heightened anxiety levels and substantial psychological stress [93,94]. Consequently, Chinese college students may frequently turn to mobile phones as a way of escapism, seeking solace from the stresses and demands of reality.

### 4.1 Limitations and directions for future research

This study acknowledges some potential limitations. Firstly, meta-analysis is a dynamic and continual process, and the conclusions drawn from it can evolve with time, this is especially relevant in cross-temporal meta-analysis. Secondly, positive, statistically significant studies have a better chance of being published and cited, but negative findings can easily be ignored [95]. Thirdly, to uphold the quality and credibility of the study, only chose core English and core Chinese journals in the study search and did not cover grey literature, which may with a trade-off in terms of comprehensiveness. Fourthly, except for gender differences, there may be other characteristic differences in mobile phone addiction among Chinese college students, such as major, grade, and class position. However, due to the limitations of the basic original studies, there is no uniform approach and report to these characteristics, and this study was unable to delve deeper into other characteristic differences in mobile phone addiction among Chinese college students. Finally, the results of this cross-temporal meta-analysis reflect only the rising trend of mobile phone addiction in Chinese college students, and it remains unclear whether these findings apply to other countries.

Despite these objective limitations, the development trend of mobile phone addiction in Chinese college students has been confirmed to be rising over the years. To expand the research related to mobile phone addiction among college students in China, academics should shift their focus from the rising trend of mobile phone addiction among college students to why it is rising, and should also focus on the differences in group characteristics of mobile phone addiction among Chinese college students, which is conducive to the construction of targeted prevention strategies. Although this study has explored the factors correlated with mobile phone addiction among college students, there is still a lack of reliable evidence to directly prove causality, future research can further cross-validate the findings of this study by using longitudinal designs and time-series data. Additionally, scholars from other countries can undertake further studies based on this study in the future to validate the applicability of these findings in diverse cultural contexts and broaden the implication scope of this study.

## 5 Conclusion

From a cross-temporal meta-analysis perspective, the present study reveal a rising trend of mobile phone addiction among Chinese college students over the last decade, and discuss the three important rising periods. Further research confirms the existence of gender differences in mobile phone addiction among Chinese college students, and shedding light on the close correlation between negative emotions with mobile phone addiction in this demographic. In addition, based on the officially published data on social indicators found that the National internet penetration rate and the National GDP index serve as significant predictors of mobile phone addiction among Chinese college students.

Mobile phones with powerful functions have become the main medium for surfing the Internet. Under the great temptation of the Internet world, excessive use of mobile phones can easily lead to mobile phone addiction [96]. Chinese college students should exercise self-discipline to avoid being ensnared by mobile phone addiction due to excessive immersion in the Internet world. In addition, faced with the stresses of daily life, challenging interpersonal relationships, and swift changes in society, Chinese college students often experience stress, anxiety and depression. Turning to mobile phones for comfort has become a habitual way for them to escape from reality, leading to mobile phone addiction [97]. However, mobile phone addiction only provides temporary psychological comfort and in the long term, it can create a harmful cycle that intensifies these negative emotions [98]. To overcome mobile phone addiction, Chinese college students need to abandon the mentality of escapism, enhance their resilience, and confront stresses and challenges directly.

## Funding statement

This research did not receive any specific grant from funding.

## Data availability statement

The data use in this study not been deposited into a publicly available repository. Data will be made available on request.

## Ethics statement

Not applicable.

## CRediT authorship contribution statement

**Chunwei Lyu:** Writing – original draft, Validation, Software, Methodology, Data curation. **Zixuan Cao:** Validation, Software, Methodology, Data curation. **Zun Jiao:** Writing – review & editing, Validation, Supervision, Resources, Project administration.

## Declaration of competing interest

The authors declare that they have no known competing financial interests or personal relationships that could have appeared to influence the work reported in this paper.

## References

[1] CNNIC (2023).

[2] Billieux J., Maurage P., Lopez-Fernandez O., Kuss D.J., Griffiths M.D. (2015). Can disordered mobile phone use be considered a behavioral addiction? An update on current evidence and a comprehensive model for future research. Current Addiction Reports.

[3] Zheng X.B., Lee M.K.O. (2016). Excessive use of mobile social networking sites: negative consequences on individuals. Comput. Hum. Behav..

[4] Seo D.G., Park Y., Kim M.K., Park J. (2016). Mobile phone dependency and its impacts on adolescents' social and academic behaviors. Comput. Hum. Behav..

[5] Mei S.L., Hu Y.Y., Wu X.G., Cao R.L., Kong Y.X., Zhang L.W., Lin X.L., Liu Q., Hu Y.C., Li L. (2023). Health risks of mobile phone addiction among college students in China. Int. J. Ment. Health Addiction.

[6] Zirek E., Mustafaoglu R., Yasaci Z., Griffiths M.D. (2020). A systematic review of musculoskeletal complaints, symptoms, and pathologies related to mobile phone usage. Musculoskeletal Science and Practice.

[7] Chen G., Lyu C. (2024). The relationship between smartphone addiction and procrastination among students: a systematic review and meta-analysis. Pers. Indiv. Differ..

[8] Buctot D.B., Kim N., Kim S.H. (2023). Comparing the mediating effect of adolescent lifestyle profiles on the relationship between smartphone addiction and health-related quality of life among male and female senior high school students in the Philippines. Int. J. Ment. Health Addiction.

[9] Lu G.L., Ding Y.M., Zhang Y.M., Huang H.T., Liang Y.P., Chen C.R. (2021). The correlation between mobile phone addiction and coping style among Chinese adolescents: a meta-analysis. Child Adolesc. Psychiatr. Ment. Health.

[10] Hao Z.J., Jin L.Y., Li Y., Akram H.R., Saeed M.F., Ma J., Ma H.B., Huang J.Z. (2019). Alexithymia and mobile phone addiction in Chinese undergraduate students: the roles of mobile phone use patterns. Comput. Hum. Behav..

[11] Abbasi G.A., Jagaveeran M., Goh Y.N., Tariq B. (2021). The impact of type of content use on smartphone addiction and academic performance: physical activity as moderator. Technol. Soc..

[12] Cain J. (2018). It's time to confront student mental health issues associated with smartphones and social media. Am. J. Pharmaceut. Educ..

[13] Xie G.M., Wu Q., Guo X.H., Zhang J.P., Yin D.H. (2023). Psychological resilience buffers the association between cell phone addiction and sleep quality among college students in Jiangsu Province, China. Front. Psychiatr..

[14] Wang J., Hao Q.H., Peng W., Tu Y., Zhang L., Zhu T.M. (2023). Relationship between smartphone addiction and eating disorders and lifestyle among Chinese college students. Front. Public Health.

[15] Ivanova A., Gorbaniuk O., Blachnio A., Przepiórka A., Mraka N., Polishchuk V., Gorbaniuk J. (2020). Mobile phone addiction, phubbing, and depression among men and women: a moderated mediation analysis. Psychiatr. Q..

[16] Chen Y., Xue Y.K., Lian S.L., Gu C.H., Dai H.B. (2018). The impact of a family's socioeconomic status on mobile phone addiction: the mediating effect of subjective well-being. Chinese Journal of Special Education.

[17] Wang H., Huang H., Wu H.M. (2014). Relationship between personality and mobile phone addiction: a mediating role of social anxiety. Chin. J. Clin. Psychol..

[18] Li Y., Li G.X., Liu L., Wu H. (2020). Correlations between mobile phone addiction and anxiety, depression, impulsivity, and poor sleep quality among college students: a systematic review and meta-analysis. Journal of Behavioral Addictions.

[19] Bian M.W., Leung L. (2015). Linking loneliness, shyness, smartphone addiction symptoms, and patterns of smartphone use to social capital. Soc. Sci. Comput. Rev..

[20] Gutiérrez J.D.-S., de Fonseca F.R., Rubio G. (2016). Cell-phone addiction: a review. Front. Psychiatr..

[21] Hedges L.V. (1982). Estimation of effect size from a series of independent experiments. Psychol. Bull..

[22] Huang H., Niu L.Y., Zhou C.Y., Wu H.M. (2014). Reliability and validity of mobile phone addiction index for Chinese college students. Chin. J. Clin. Psychol..

[23] Leung L. (2008). Linking psychological attributes to addiction and improper use of the mobile phone among adolescents in Hong Kong. J. Child. Media.

[24] Moher D., Liberati A., Tetzlaff J., Altman D.G., Grp P. (2014). Preferred reporting items for systematic reviews and meta-analyses: the PRISMA statement. Rev. Española Nutr. Humana Dietética.

[25] Cumpston M., Li T.J., Page M.J., Chandler J., Welch V.A., Higgins J.P.T., Thomas J. (2019). Updated guidance for trusted systematic reviews: a new edition of the Cochrane Handbook for Systematic Reviews of Interventions. Cochrane Database Syst. Rev..

[26] Yuan M.L., Spadaro G., Jin S.X., Wu J.H., Kou Y., Van Lange P.A.M., Balliet D. (2022). Did cooperation among strangers decline in the United States? A cross-temporal meta-analysis of social dilemmas (1956-2017). Psychol. Bull..

[27] Institute J.B. (2017).

[28] Jefferson L., Golder S., Heathcote C., Avila A.C., Dale V., Essex H., van der Feltz Cornelis C., McHugh E., Moe-Byrne T., Bloor K. (2022). GP wellbeing during the COVID-19 pandemic: a systematic review. Br. J. Gen. Pract..

[29] Jiao Z., Chen Y., Lyu C. (2024). Factors correlated with personal growth initiative among college students: a meta-analysis. Heliyon.

[30] Martins J.N., Marques D., Silva E.J.N.L., Caramês J., Mata A., Versiani M.A. (2020). Influence of demographic factors on the prevalence of a second root canal in mandibular anterior teeth–a systematic review and meta-analysis of cross-sectional studies using cone beam computed tomography. Arch. Oral Biol..

[31] Hedges L.V. (1981). Distribution theory for Glass's estimator of effect size and related estimators. J. Educ. Stat..

[32] Higgins J.P., Thompson S.G., Deeks J.J., Altman D.G. (2003). Measuring inconsistency in meta-analyses. BMJ.

[33] Deeks J.J., Macaskill P., Irwig L. (2005). The performance of tests of publication bias and other sample size effects in systematic reviews of diagnostic test accuracy was assessed. J. Clin. Epidemiol..

[34] Huang H., Hou J.X., Yu L., Zhou C.Y. (2014). A comparative analysis on mental health of college students with internet addiction and mobile phone addiction. Chin J Sch Health.

[35] Liu W.L., Cai T.S. (2015). The mediating effect of loneliness between social support and phone addiction tendency. Chin. J. Clin. Psychol..

[36] Wang Y.Q., Zhang Y. (2015). Relation of mobile phone addiction to perceived social support and subjective well-being in college students, Chinese Mental. Health Journal.

[37] Zhang Y., Zhou Y.G., Pei T. (2015). Mediating effect of loneliness on relationship between interpersonal adaptation and mobile phone addiction in college students, Chinese Mental. Health Journal.

[38] Deng Z.J., Huang H., Gui Y.F., Niu L.Y., Zhou C.Y. (2015). Mobile phone dependence, parenting style and subjective well-being in college students, Chinese Mental. Health Journal.

[39] Huang H., Li C.J., Gui Y.F., Zhou C.Y., Wu H.M., Zhang J.Y. (2015). Undergraduates' impulsivity and mobile phone addiction: a mediating role of alienation. Chin. J. Clin. Psychol..

[40] Yao M.P., Jia Z.B., Chen X., Qiao S.S. (2016). Meaning in life mediates the relationship between boredom and mobile phone dependency behavior among college students. Chin J Sch Health.

[41] Lian L., You X.Q., Huang J., Yang R.J. (2016). Who overuses Smartphones? Roles of virtues and parenting style in Smartphone addiction among Chinese college students. Comput. Hum. Behav..

[42] Zhou S.Q., Luo S.R., Li Z.M., Ye M.L., Li Y.N. (2017). Study on mobile phone usage and its influencing factors among medical university students in Nanning. Chin. J. Health Statistics.

[43] Hu Y., Huang H., Zhang Y.Q., Zhou C.Y. (2017). The mediating effect of negative emotions between mobile phone dependence and cognitive failure. Chin. J. Clin. Psychol..

[44] Ni L.Y., Deng W.G. (2017). An empirical study of mobile phone internet dependence, self-esteem and interpersonal competence among senior college students. EDUCATION RESEARCH MONTHLY.

[45] Zhang Y., Lei T.T., Wang H.R., Ding L., Li D.Y., Zhou Y.G. (2018). Relationship between parent-child attachment and negative affect in college students: multiple mediation effects of interpersonal adaptation and mobile phone addiction. Mod. Prev. Med..

[46] Gao Y.Y., Chen Z., Zhang X., Li J.Y. (2018). Relationship of mobile phone dependence, resilience and emotion among college students. Mod. Prev. Med..

[47] Zhao Y. (2019). Relationship between mobile phone dependence and perfectionism among college students in Nanjing: the mediating effect of social anxiety. Mod. Prev. Med..

[48] Zhu J.J., Xie R.Q., Chen Y.Y., Zhang W. (2019). Relationship between parental rejection and problematic mobile phone use in Chinese university students: mediating roles of perceived discrimination and school engagement. Front. Psychol..

[49] Xie F., Song L.P. (2019). Influence of emotional and cognitive appraisal orientation on smartphone addiction among medical students. Journal of Nursing Science.

[50] Li W.F., Zhang X.T., Chu M.H., Li G.Y. (2020). The impact of adverse childhood experiences on mobile phone addiction in Chinese college students: a serial multiple mediator model. Front. Psychol..

[51] Chen J.J., Li H.P., Yang Y.J., Wang Q.L., Hong L. (2020). Mediation effect of psychological resilience in the relationship between mobile phone addiction and mental health in college students. Mod. Prev. Med..

[52] Qiu L., Zhou X.H., Feng C.X., Li Y.J., Yang Q. (2020). The mediating role of resilience in the relationship between mobile phone addiction and social support among medical students. The Chinese Health Service Management.

[53] Zhao C.J., Xu H.H., Lai X.Y., Yang X., Tu X.L., Ding N.N., Lv Y.J., Zhang G.H. (2021). Effects of online social support and perceived social support on the relationship between perceived stress and problematic smartphone usage among Chinese undergraduates. Psychol. Res. Behav. Manag..

[54] Li L., Griffiths M.D., Mei S.L., Niu Z.M. (2020). Fear of missing out and smartphone addiction mediates the relationship between positive and negative affect and sleep quality among Chinese university students. Front. Psychiatr..

[55] Yang X.F., Li X.F., Hu P. (2020). Trait procrastination and mobile phone addiction in college students: the mediating role of negative affect. Chin. J. Clin. Psychol..

[56] Li X.W., Feng X.C., Xiao W.L., Zhou H. (2021). Loneliness and mobile phone addiction among Chinese college students: the mediating roles of boredom proneness and self-control. Psychol. Res. Behav. Manag..

[57] Liu Q.Q., Yang X.J., Zhu X.W., Zhang D.J. (2021). Attachment anxiety, loneliness, rumination and mobile phone dependence: a cross-sectional analysis of a moderated mediation model. Curr. Psychol..

[58] Zhao J.Y., Cen Y., Yang J.M., Liu C., Li Y.J., Ren Z., Xiao Y., He J.L., Luo J., Zhong Y.L., Luo W.X., Wu J., Luo J.M. (2022). Prevalence and correlates of sleep quality in the Chinese college students with migraine: a cross-sectional study. Front. Behav. Neurosci..

[59] Chen J.B., Shen X.Y., Li L.Y., Han Y., Zhu X.Z. (2021). Measurement invariance of the mobile phone addiction index across gender in college students. Chin. J. Clin. Psychol..

[60] Huang F., Guo F., Ding Q., Hong J.Z. (2021). Social anxiety and mobile phone addition in college students: the influence of cognitive failure and regulatory emotional self-efficacy. Chin. J. Clin. Psychol..

[61] Li L., Niu Z.M., Mei S.L., Griffiths M.D. (2022). A network analysis approach to the relationship between fear of missing out (FoMO), smartphone addiction, and social networking site use among a sample of Chinese university students. Comput. Hum. Behav..

[62] Wang L.F., Wang R.Y. (2022). Relationship between nervousness and smartphone dependence among college students: the mediating effects of mental health and expressive suppression. Chinese Journal of Health Psychology.

[63] Jiang W.N., Luo J., Guan H.N., Jiang F., Tang Y.L. (2022). Problematic mobile phone use and life satisfaction among university students during the COVID-19 pandemic in Shanghai, China. Front. Public Health.

[64] Liu F., Xu Y.A., Yang T.S., Li Z.H., Dong Y.K., Chen L., Sun X.H. (2022). The mediating roles of time management and learning strategic approach in the relationship between smartphone addiction and academic procrastination. Psychol. Res. Behav. Manag..

[65] Li X.D., Long S.L. (2022). Research on the prevalence, mental health and influencing factors of mobile phone addiction among medical college students in the post-epidemic era. Chinese Journal of Health Psychology.

[66] Cheng P., Zhang H., Ge W.X., Liu Y. (2023). The effect of alexithymia on mobile phone addiction: the sequential mediating effect of negative evaluation fear and self-control. Chin. J. Clin. Psychol..

[67] Jiang H.B., Liang H.Y., Li B., Tuo A. (2023). Alexithymia and mobile phone addiction among college students: mediation by boredom proneness and anxiety. J. Psychol. Afr..

[68] Wei Z.F. (2023). Effect of self-control on academic procrastination in college students: the chain mediating role of mobile phone addiction and learning engagement. Chin. J. Clin. Psychol..

[69] Zhang H., Lin Y., Yang Y.Q., Zhang J.Q. (2023). Effect of alexithymia on mobile phone addiction among vocational college students: the mediation of negative evaluation fear and gender regulation. Chinese Journal of Health Psychology.

[70] Lee C., Kim O. (2017). Predictors of online game addiction among Korean adolescents. Addiction Res. Theor..

[71] Haberlin K.A., Atkin D.J. (2022). Mobile gaming and Internet addiction: when is playing no longer just fun and games?. Comput. Hum. Behav..

[72] Yao S., Chen Y. (2022). Reconstructing history and culture in game discourse: a linguistic analysis of heroic stories in honor of kings. Game. Cult..

[73] Li F., Song L. (2023). Video game music and cultural dissemination: a study of honour of kings as a MOBA game. Galactica Media: Journal of Media Studies.

[74] Scherr S., Wang K. (2021). Explaining the success of social media with gratification niches: motivations behind daytime, nighttime, and active use of TikTok in China. Comput. Hum. Behav..

[75] Zhang N., Hazarika B., Chen K., Shi Y. (2023). A cross-national study on the excessive use of short-video applications among college students. Comput. Hum. Behav..

[76] Zhou B. (2019). Fear of missing out, feeling of acceleration, and being permanently online: a survey study of university students' use of mobile apps in China. Chin. J. Commun..

[77] Bonanno P., Kommers P.A. (2005). Gender differences and styles in the use of digital games. Educ. Psychol..

[78] Hasan B. (2010). Exploring gender differences in online shopping attitude. Comput. Hum. Behav..

[79] Smit M., Bilos A., Turkalj D. (2021). Internet usage and related behavior patterns of primary school children: perceived differences between girls and BOYS IN Croatia. Ekonomski Vjesnik.

[80] Andrejek N. (2021). Girls' night out: the role of women-centered friendship groups in university hookup culture. Socio. Forum.

[81] Ho H.C., Awan M.A. (2019). The gender effect on consumer attitudes toward payment methods: the case of online Chinese customers. J. Internet Commer..

[82] Chang S.M., Hsieh G.M.Y., Lin S.S.J. (2018). The mediation effects of gaming motives between game involvement and problematic Internet use: escapism, advancement and socializing. Comput. Educ..

[83] Simon R.M., Wagner A., Killion B. (2017). Gender and choosing a STEM major in college: femininity, masculinity, chilly climate, and occupational values. J. Res. Sci. Teach..

[84] Kugler A.D., Tinsley C.H., Ukhaneva O. (2021). Choice of majors: are women really different from men?. Econ. Educ. Rev..

[85] Stankovic M., Nesic M., Cicevic S., Shi Z.H. (2021). Association of smartphone use with depression, anxiety, stress, sleep quality, and internet addiction. Empirical evidence from a smartphone application. Pers. Indiv. Differ..

[86] Yeh C.W., Chen T.Y. (2023). The role of online game usage in the relationship between initial daily negative moods and subsequent positive moods: the moderating role of hedonistic motivation. Curr. Psychol..

[87] Huang L., Zhang T. (2022). Perceived social support, psychological capital, and subjective well-being among college students in the context of online learning during the COVID-19 pandemic. Asia-Pacific Education Researcher.

[88] Ye Z., Yang X.Y., Zeng C.B., Wang Y.Y., Shen Z.J., Li X.M., Lin D.H. (2020). Resilience, social support, and coping as mediators between COVID-19-related stressful experiences and acute stress disorder among college students in China. Applied Psychology-Health and Well Being.

[89] Shi Y.X., Koval P., Kostakos V., Goncalves J., Wadley G. (2023). Instant Happiness: smartphones as tools for everyday emotion regulation. Int. J. Hum. Comput. Stud..

[90] Li J., Lepp A., Barkley J.E. (2015). Locus of control and cell phone use: implications for sleep quality, academic performance, and subjective well-being. Comput. Hum. Behav..

[91] Guo C.B., Wan B.S. (2022). The digital divide in online learning in China during the COVID-19 pandemic. Technol. Soc..

[92] Cheng C., Li A.Y.L. (2014). Internet addiction prevalence and quality of (real) life: a meta-analysis of 31 nations across seven world regions. Cyberpsychol., Behav. Soc. Netw..

[93] Cheng C., Lau H.P.B., Chan M.P.S. (2014). Coping flexibility and psychological adjustment to stressful life changes: a meta-analytic review. Psychol. Bull..

[94] Greenfield P.M. (2009). Linking social change and developmental change: shifting pathways of human development. Dev. Psychol..

[95] Guyatt G.H., Oxman A.D., Montori V., Vist G., Kunz R., Brozek J., Alonso-Coello P., Djulbegovic B., Atkins D., Falck-Ytter Y., Williams J.W., Meerpohl J., Norris S.L., Akl E.A., Schünemann H.J. (2011). GRADE guidelines: 5. Rating the quality of evidence-publication bias. J. Clin. Epidemiol..

[96] Roberts J.A., Pullig C., Manolis C. (2015). I need my smartphone: a hierarchical model of personality and cell-phone addiction. Pers. Indiv. Differ..

[97] Liu Q.-Q., Zhang D.-J., Yang X.-J., Zhang C.-Y., Fan C.-Y., Zhou Z.-K. (2018). Perceived stress and mobile phone addiction in Chinese adolescents: a moderated mediation model. Comput. Hum. Behav..

[98] Zhao C., Ding H., Du M., Yu Y., Chen J.H., Wu A.M.-S., Wang D.B., Du M., Chen Y., Luo Q. (2024). The vicious cycle between loneliness and problematic smartphone use among adolescents: a random intercept cross-lagged panel model. J. Youth Adolesc..

